# Fatty Acid Binding Protein 4 Is Associated with Carotid Atherosclerosis and Outcome in Patients with Acute Ischemic Stroke

**DOI:** 10.1371/journal.pone.0028785

**Published:** 2011-12-09

**Authors:** Sverre Holm, Thor Ueland, Tuva B. Dahl, Annika E. Michelsen, Mona Skjelland, David Russell, Ståle H. Nymo, Kirsten Krohg-Sørensen, Ole Petter Clausen, Dan Atar, James L. Januzzi, Pål Aukrust, Jesper K. Jensen, Bente Halvorsen

**Affiliations:** 1 Research Institute for Internal Medicine, Oslo University Hospital Rikshospitalet, Oslo, Norway; 2 Department of Neurology, Oslo University Hospital Rikshospitalet, Oslo, Norway; 3 Department of Thoracic and Cardiovascular Surgery, Oslo University Hospital Rikshospitalet, Oslo, Norway; 4 Institute of Pathology, Oslo University Hospital Rikshospitalet, Oslo, Norway; 5 Section of Clinical Immunology and Infectious Diseases, Oslo University Hospital Rikshospitalet, Oslo, Norway; 6 Division of Cardiology, Oslo University Hospital Ullevål, Oslo, Norway; 7 Faculty of Medicine, University of Oslo, Oslo, Norway; 8 Cardiology Division, Massachusetts General Hospital, Boston, Massachusetts, United States of America; 9 Department of Cardiology, Odense University Hospital, Odense, Denmark; Julius-Maximilians-Universität Würzburg, Germany

## Abstract

**Background and Purpose:**

Fatty acid binding protein 4 (FABP4) has been shown to play an important role in macrophage cholesterol trafficking and associated inflammation. To further elucidate the role of FABP4 in atherogenesis in humans, we examined the regulation of FABP4 in carotid atherosclerosis and ischemic stroke.

**Methods:**

We examined plasma FABP4 levels in asymptomatic (n = 28) and symptomatic (n = 31) patients with carotid atherosclerosis, as well as in 202 subjects with acute ischemic stroke. In a subgroup of patients we also analysed the expression of FABP4 within the atherosclerotic lesion. In addition, we investigated the ability of different stimuli with relevance to atherosclerosis to regulate FABP4 expression in monocytes/macrophages.

**Results:**

FABP4 levels were higher in patients with carotid atherosclerosis, both systemically and within the atherosclerotic lesion, with particular high mRNA levels in carotid plaques from patients with the most recent symptoms. Immunostaining of carotid plaques localized FABP4 to macrophages, while activated platelets and oxidized LDL were potent stimuli for FABP4 expression in monocytes/macrophages *in vitro*. When measured at the time of acute ischemic stroke, high plasma levels of FABP4 were significantly associated with total and cardiovascular mortality during follow-up, although we did not find that addition of FABP4 to the fully adjusted multivariate model had an effect on the prognostic discrimination for all-cause mortality as assessed by c-statistics.

**Conclusions:**

FABP4 is linked to atherogenesis, plaque instability and adverse outcome in patients with carotid atherosclerosis and acute ischemic stroke.

## Introduction

Atherosclerosis is a chronic disease characterized by two fundamental hallmarks: lipid accumulation and inflammation. [1] The interaction between these two processes defines the principal pathogenesis of atherosclerosis, and distinguishes it from other chronic inflammatory disorders. Carotid atherosclerosis may result in cerebral embolization and symptoms of cerebral ischemia leading to transient ischemic attack (TIA) or stroke. While the interaction between inflammation and lipids leads to progression of carotid plaques with the development of symptomatic lesions; [1], [2] the molecular mechanisms for these events are not fully understood.

Fatty acid binding proteins (FABPs) are cytosolic proteins that function as lipid chaperones and are involved in lipid signalling cascades. [3] Amongst the FABPs, FABP4 (aP2) is of special interest in atherogenesis. While originally described as an adipose tissue protein, [4], [5] recent work has shown a pivotal role for FABP4 in macrophages in relation to cholesterol trafficking and inflammation. [6], [7] In line with this, total or macrophage-specific FABP4 (aP2)-deficiency has been shown to protect against atherosclerosis in apolipoprotein E-deficient (ApoE^-/-^) mice, [8] and FABP4 has been suggested as a potential drug target in diseases like diabetes and atherosclerosis. [6] Two very recent studies have shown enhanced FABP4 expression within human carotid atherosclerotic lesions in association with poor prognosis, [9], [10] but the role and regulation of FABP4 in clinical atherosclerosis is still unclear.

To further elucidate the role of FABP4 in atherogenesis in humans, we examined the regulation of FABP4 in atherosclerosis and ischemic stroke in three different ways: (i) In a cross-sectional study of patients with symptomatic and asymptomatic carotid plaques we analyzed FABP4 levels in plasma and plaque samples. (ii) In an *in vitro* study, we examined the regulation of FABP4 in monocytes/macrophages. (iii) In a separate sub-study, we examined the association between plasma levels of FABP4 in acute ischemic stroke and mortality during long-term follow-up.

## Materials and Methods

### Ethics

The study was approved by the Regional Committee of Medical and Health Research Ethics (REK) in Eastern Norway and signed informed consent was obtained from all individuals.

### Patients and controls

#### Cross-sectional study

Patients with high-grade internal carotid stenoses (≥70%) treated with carotid endarterectomy or carotid angioplasty with stenting were consecutively recruited to the study. Patients were classified as asymptomatic and symptomatic according to the absence or presence of clinical symptoms such as stroke, TIA or amaurosis fugax ipsilateral to the stenotic internal carotid artery within the past 6 months. Carotid stenoses were diagnosed and classified by precerebral color Duplex ultrasound and CT angiography according to consensus criteria. [11] Asymptomatic carotid stenoses were detected during clinical examinations of patients with coronary artery disease (CAD), peripheral artery disease or stroke/TIA more than six months ago. The plaques were also divided into two groups (i.e., echolucent or echogenic/heterogeneous) depending on plaque echogenicity on ultrasound examination. [11] Plasma samples were analyzed from 31 symptomatic and 28 asymptomatic patients, whereas plaque mRNA expression was analyzed in 42 symptomatic and 12 asymptomatic. All patients were recruited from the same cohort, and we attempted to collect plasma and plaques from all patients, but in some cases this was not possible. For comparisons, blood samples were also collected from 18 sex- and age-matched healthy individuals recruited from the same area of Norway as the patients (eastern part). Although asymptomatic atherosclerosis can not be totally excluded, all the controls were evaluated as healthy based on clinical examination, disease history and analyses of C-reactive protein (CRP) and lipid parameters (Table 1).

**Table 1:** Baseline variables in patients according to symptomatic# and asymptomatic carotid plaques (n = 59) and healthy controls (n = 18).

	Symptomatic plaques (n = 31)	Asymptomatic plaques (n = 28)	p	Controls (n = 18)
Age, year	66.8 (8.8, range 50–83)	65.3 (9.4, range 44–81)	.533	59 (6, range 47–70)
Male sex*	21 (67.7)	19 (67.9)	.992	17 (85)
Current smoking^*^	20 (64.5)	16 (57.1)	.562	2 (10)
Degree of stenoses, %	80 (60–95)	80 (60–95)	.724	n.d.
Echolucent carotid plaque*	12 (38.7)	8(28.6)	.411	n.d.
Ipsilateral ischemia on cerebral MRI* (n = 47)	23 (74.2)	18 (64.3)	.887	n.d.
Body mass index, kg/m^2^	25 (20–36)	27 (19–35)	.164	n.d.
Systolic blood pressure,mmHg	152 (110–200)	149 (111–176)	.323	n.d.
Diastolic blood pressure, mmHg	79.5 (32–101)	77.5 (49–99)	.222	n.d.
Statin treatment^*^	26 (83.9)	25 (89.3)	.544	0
Aspirin treatment^*^	27 (87.1)	23 (82.1)	.587	0
FABP4 ng/ml	32.3 (14.4 ) *(median 28.3, range 18.5–78.4)*	29.1 (7.5) *(median 29.2, range 14.1–43.3*)	.294	19.0 (4.3) *(median 18.5, range 7.2–25.1*)
CRP, mg/l	4.1 (1.0–39.0)	5 (1.0–28.0)	.724	1.01 (0.3)
Total leukocyte count, 10^9^/l	8.1 (2.1)	7.1 (1.6)	.044	n.d.
Cholesterol, mmol/l	4.5 (0.9)	4.2 (0.91)	.154	4.3 (0.6)
HDL cholesterol, mmol/l (n = 88)	1.4 (0.5)	1.3 (0.4)	.584	1.4 (0.3)
LDL (n = 78)	2.8 (0.82)	2,5 (0.75)	.189	2.6 (0.6)
HbA1c, %	6.0 (0.9)	6.0 (1.0)	.839	n.d.
Platelet count, 10^9^/l	257 (73.4)	266 (47.5)	.593	n.d.

P-value: Symptomatic patients vs asymptomatic.

# Clinical symptoms include stroke, TIA or amaurosis fugax ipsilateral to the stenotic internal carotid artery within the last 6 months. Values are mean (SD) in normally distributed data, median (range) in skewed data or *numbers (percentages).

#### Patients with acute stroke – longitudinal follow-up study

Between August 2003 and October 2004, 790 patients with acute ischemic stroke admitted to the Department of Neurology, Odense University Hospital, Odense, Denmark were consecutively screened for inclusion into the study. [12] Patients with overt ischemic heart disease (n = 177) (i.e., any prior myocardial infarction, stable or unstable angina pectoris, pathological Q-waves on the baseline electrocardiogram, previous coronary angioplasty or coronary bypass surgery), patients with current atrial fibrillation (n = 132), patients with onset of stroke symptoms >7 days before admission (n = 75) were not included. In addition, 70 patients were excluded because of lack of compliance, 20 patients were transferred to other hospitals, in 10 patients the stroke diagnosis was revised after re-evaluation and 15 patients were excluded of other reasons. Finally, 47 patients were unwilling to participate, rendering 244 patients eligible for inclusion in the study, of which plasma samples were available from 202 patients (Table 2). Clinical evaluations were performed by a senior neurologist that was blinded to the biomarkers, and the presence of intracerebral or subarachnoid hemorrhage was ruled out by computed tomography at the time of admission. Patient demographics along with past and present clinical history including medication were obtained by interviewing the patient and from medical records. Relatives provided this information if the patient was unable to take part in the interview. Renal failure was defined as plasma creatinine >120 µmol/L. Heart failure (HF) was considered present if the patients had a left ventricular ejection fraction <50% and/or if a patient had previously been given the diagnosis of HF by a physician. Stroke severity was assessed using the Scandinavian Stroke Scale (SSS). [13] Patients with concomitant inflammatory disease (e.g., infection or autoimmune disorders), malignancies or liver disease were excluded from follow-up.

**Table 2:** Baseline characteristics of study patients.

Variable	
Age, year	69±13
Men	107 (53%)
History	
Previous ischemic stroke	42 (21%)
Hypertension	111 (55%)
Diabetes Mellitus	24 (12%)
Lacunar infarction	97 (48%)
Total anterior circulation infarction	10 (5%)
Partial anterior circulation infarction	63 (31%)
Posterior circulation infarction	28 (14%)
Smoking	103 (51%)
Prior heart and/or renal failure	26 (13%)
Physical examination	
Pulse	78±15
Systolic blood pressure, mm Hg	175±15
Diastolic blood pressure, mm Hg	93±17
Scandinavian Stroke Scale score	47 (36–53)
Laboratory findings	
Troponin T >0.03 g/L	14 (7%)
Hemoglobin, g/dL	8.58±1.06
Creatinine, mg/dL	91 (80–103)
C-reactive protein, mg/L	5 (5–14)

Proportions are given as no. and (%). Continuous variables are given as mean ± standard deviation or median and interquartile range depending on distribution.

### Blood Sampling Protocol

Peripheral venous blood was drawn into pyrogen-free EDTA tubes which were immediately immersed in melting ice and centrifuged at 2500 *g* for 25 minutes within 20 minutes to obtain platelet-poor plasma. All samples were stored at –80°C; samples were thawed only once before analyses.

### Carotid Endarterectomy Specimens

Atherosclerotic carotid plaques were retrieved from patients during carotid endarterectomy. Plaques that were used for RNA extraction were rapidly frozen in liquid nitrogen. Plaques that were used for histological analyses were put in 4% phosphate buffered-formalin for 48 hours and then embedded in paraffin.

### Real-Time Quantitative Reverse Transcription Polymerase Chain Reaction

Total RNA was isolated from frozen THP-1 monocytes and carotid tissue with the use of RNeasy spin columns (QIAGEN, Hilden, Germany) and stored at –80°C until further analysis. cDNA was synthesized using high-capacity cDNA archive kit (Applied Biosystems, Foster City, CA). Primers for FABP4 (forward primer [FP]: 5′-TTGACGAAGTCACTGCAGATGA-3′ and reverse primer [RP]: 5′-CAGGACACCCCCATCTAAGGT-3′), CD68 (FP 5′-ATCCCCACCTGCTTCTCTCA-3′ and RP 5′-GAGGTCCTGCATGAATCCAAA-3′) and adipose differentiation-related protein (ADFP) (FP 5′-GAATCAGCCATCAACTCAGATTGT-3′ and RP 5′-AGTAGTCGTCACAGCATCTTTTGC-3′) were designed with the use of Primer Express software version 3.0 (Applied Biosystems). Quantification of mRNA was performed using the ABI Prism 7500 (Applied Biosystems). SYBR Green assay was performed with the Power SYBR Green Master Mix (Applied Biosystems). Gene expression of the reference gene β-actin was used for normalization.

### Immunohistochemistry

Sections (5 µm) of paraffin embedded atherosclerotic plaques were treated with 0.5% H_2_0_2_, followed by high-temperature unmasking in citrate-buffer (pH 6), blocked with 0.5% bovine serum albumin (BSA) and then incubated with primary antibody (rabbit anti-human FABP4; Abcam, Cambridge, UK) for one hour at room temperature. After washing, the slides were incubated for 30 minutes with peroxidase-conjugated secondary antibodies (Impress-Vector, Vector laboratories, Burlingame, CA), rinsed and developed with chromogen for immunoperoxidase staining (DAB Plus, Vector laboratories) for 7 minutes. The sections were counterstained with Hematoxylin. Omission of the primary antibody served as a negative control.

### Immunfluorescence

Paraffin-embedded sections (5 µm) of atherosclerotic carotid plaques were exposed to high-temperature unmasking (citrate-buffer, pH 6), blocked in 0.5% BSA and incubated over night at 4°C with rabbit anti-human FABP4 (Abcam) and mouse anti-human CD68 (Dako, Glostrup, Denmark). The sections were counterstained with Alexa Fluor 488-conjugated goat anti-rabbit IgG and Alexa Fluor 568-conjugated donkey anti-mouse IgG (both from Invitrogen), respectively. Nuclei were stained with diamidino-2-phenylindole (DAPI) (Slow Fade Gold antifade reagent, Invitrogen). Fluorescent images were obtained on a Nikon Eclipse E400 microscope with the 40× objective.

### Cell Culture Experiments

The *human monocytic cell line THP-1* (American Type Culture Collection, Rockville, MD) was cultured for 4 days in RPMI 1640 (PAA laboratories, Pasching, Austria), supplemented with 10% fetal bovine serum (Gibco, Grand Island, NY), with and without recombinant human tumor necrosis factor α (rhTNFα, 5 ng/ml; R&D Systems, Minneapolis, MN), before further incubation with or without lipopolysaccharide (LPS) from *E. coli* 026:B6 (5 ng/ml; Sigma, St Louis, MO), a toll-like receptor (TLR)2 agonist (Pam3Cys, 1 µg/ml; Sigma), isoproterenol (20 µmol/L, Sigma), rh-interleukin (IL)-1β (1 ng/ml, R&D Systems) and platelet releasate from un-stimulated and thrombin-activated platelets. Platelet releasate was prepared as previously described. [14] After 24 hours, cell-free supernatants were collected and stored at –80°C. In a separate experiment, THP-1 cells were differentiated into macrophages by incubation for 24 hours with phorbol myristate acetate (PMA, 100 nM; Sigma). At different time points, cells were harvested in lysisbuffer and stored at –80°C until RNA isolation. Some PMA-differentiated macrophages were further stimulated with oxidized LDL (oxLDL, 20 µg/ml, prepared as previously described), [15] with and without co-incubation with TNFα (5 ng/ml) for 18 hours before harvesting.

### Enzyme Immunoassay

FABP4 levels in plasma and cell supernatants were measured by enzyme immunoassay (EIA) (BioVendor, Modrice, Czech Republic). Plasma levels of β-thromboglobulin (β-TG) were measured by EIA from Diagnostica Stago (Asnières, France). CRP levels were determined by a high-sensitivity particle enhanced immunoturbidimetric assay on a Modular platform (Roche Diagnostic, Basel, Switzerland). The intra- and inter-assay coefficient of variation were <10% for all assays.

### Statistical Analyses

For comparisons of two groups of individuals, the Mann-Whitney *U* test was used. When more than two groups were compared, the Kruskal-Wallis test was used. If a significant difference was found, the Mann-Whitney *U* test was used to determine the differences between each pair of groups. In the *in vitr*o experiments, the Student's *t* test and One-way ANOVA were used as appropriate. Correlations were assessed by Pearson χ2. Receiver-operating characteristic (ROC) curves were established for FABP4 as a predictor of death. Kaplan-Meier analysis with log-rank test was performed to compare mortality rate in tertiles of FABP4 (comparisons pooled over strata). The plasma FABP4 levels were highly skewed and we anticipated a non-linear relationship with all-cause mortality. A restricted cubic spline analysis with five knots was also undertaken to assess linearity of risk. We chose tertiles as two groups (i.e. median) could fail to detect a non-linear association between plasma FABP4 levels and all-cause mortality, while we wanted to avoid too many small groups. Based on the Kaplan Meier curves, the association seems to be particularly strong in the third tertile. We therefore chose this cut-off for subsequent analysis. The Cox proportional hazards model was applied to assess the effect of FABP4 on survival at follow-up. In the multivariate analyses, we included parameters that were imbalanced between patients that survived and those who died (p<0.1) including age, presence of HF, SSS score, troponin T (TnT) levels >0.03 µg/L, MAP, stroke localization as well as fasting blood glucose, and mean arterial pressure (MAP) as an established risk factor for stroke. All continuous variables are expressed per SD change in the regression analysis. When testing the impact of the interaction term FABP4*SSS score, the following variables were entered forced in the first block: age, presence of HF, troponin T (TnT) levels >0.03 µg/L, fasting blood glucose, MAP and stroke localization, while SSS score, FABP4 and the interaction term SSS score*FABP4 were entered stepwise in the next block. Harrel's C-statistic was calculated for all-cause and cardiovascular (CV) mortality using the full model with and without admission levels of FABP4, and the difference between the C-statistics was estimated. To correct for over-optimism associated with validating a model in the same material from which it is developed, we implemented a jack-knife cross-validation approach, where predictions for each observation were obtained from models developed on the remaining observations. These cross-validated probabilities were used to calculate jack-knife C-statistics. In all tests, a p-value <0.05 was considered statistically significant.

## Results

### Increased plasma levels of FABP4 in patients with carotid atherosclerotic plaques

**Figure 1:**
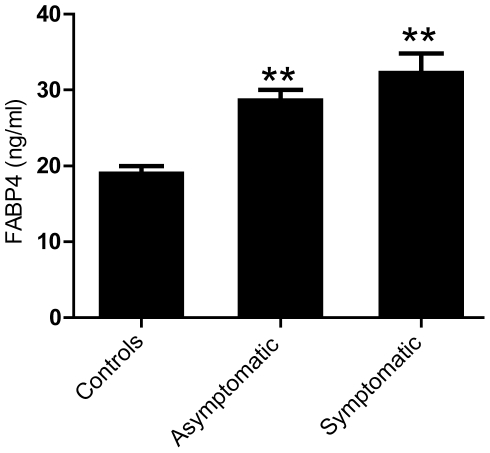
Increased plasma levels of FABP4 in patients with carotid atherosclerotic plaques. Plasma levels of FABP4 were measured by enzyme immunoassay in patients with asymptomatic (n = 28) and symptomatic (n = 31) carotid plaques and healthy controls (n = 18). Data are mean±SEM. **p<0.001 compared to controls.

Baseline characteristics of the study population (cross-sectional study) where plasma levels of FABP4 were analyzed are shown in Table 1. The study population included 40 men and 19 women, median age 67 years (range 44–83 years). As depicted in Figure 1, these 59 patients with carotid atherosclerosis had increased plasma levels of FABP4 when compared with sex- and age-matched healthy controls (n = 18), but with no differences between patients with asymptomatic (n = 28) and symptomatic (n = 31) carotid lesion (p = 0.29). The controls were comparable to the patients in relation to sex and age, but the percentage of smokers was lower in the control group. However, we found no correlation between FABP4 and smoking habits, and it is therefore unlikely that the enhanced levels of FABP4 in the patient group merely reflect an increased percentage of smokers in these individuals. Within the patient group as a whole, there was no relationship between plasma levels of FABP4 and plaque echogenicity, and FABP4 levels in patients with the most recent symptoms (<1 month) were not different from FABP4 levels in the other symptomatic patients (data not shown).

We found no significant correlations between plasma levels of FABP4 and lipid parameters in peripheral blood (triglycerides [R^2^ = 0.0071], total cholesterol [R^2^ = 0.0125], LDL cholesterol [R^2^ = 0.0069] and HDL cholesterol [R^2^ = 0.065]; p>0.1 for all comparisons). Moreover, there were no significant correlations between plasma levels of FABP4 *and* platelet counts (R^2^ = 0.0036), plasma levels of β-TG as a soluble marker of platelet activation (R^2^ = 0.0017) or CRP (R^2^  =  0.048).

### Increased mRNA expression of FABP4 in carotid atherosclerotic plaques from patients with recent symptoms

**Figure 2:**
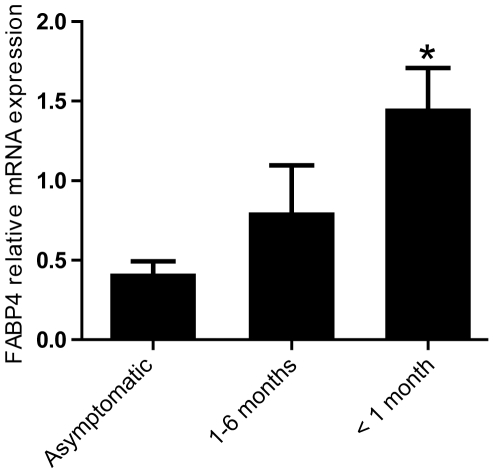
Increased FABP4 expression in atherosclerotic carotid plaques from symptomatic patients. mRNA levels of FABP4 in atherosclerotic carotid plaques were measured in 12 patients with asymptomatic carotid plaques, in 25 patients with symptoms within the last months (<1 month) and in 17 patients with symptoms 1–6 months prior to collection. mRNA levels were quantified by real-time RT-PCR. The expression of β-actin was used as endogenous control. Data are mean ± SEM. *p<0.05 compared to asymptomatic patients.

We next examined mRNA levels of FABP4 in samples obtained from asymptomatic (n = 12) and symptomatic (n = 42) carotid lesions using real-time quantitative RT-PCR. The patients with symptomatic lesion were further divided into two groups according to their latest clinical symptoms (i.e., symptoms within the last 1 month [n = 25] and symptoms within the last 1 to 6 months [n = 17]). As can be seen in Figure 2, levels of FABP4 mRNA were significantly raised in plaques from patients with the most recent symptoms (<1 month) as compared with the other patients with symptomatic carotid plaques (1–6 months) and those with asymptomatic lesions.

### FABP4 is co-localized to macrophages within carotid atherosclerotic plaques

**Figure 3:**
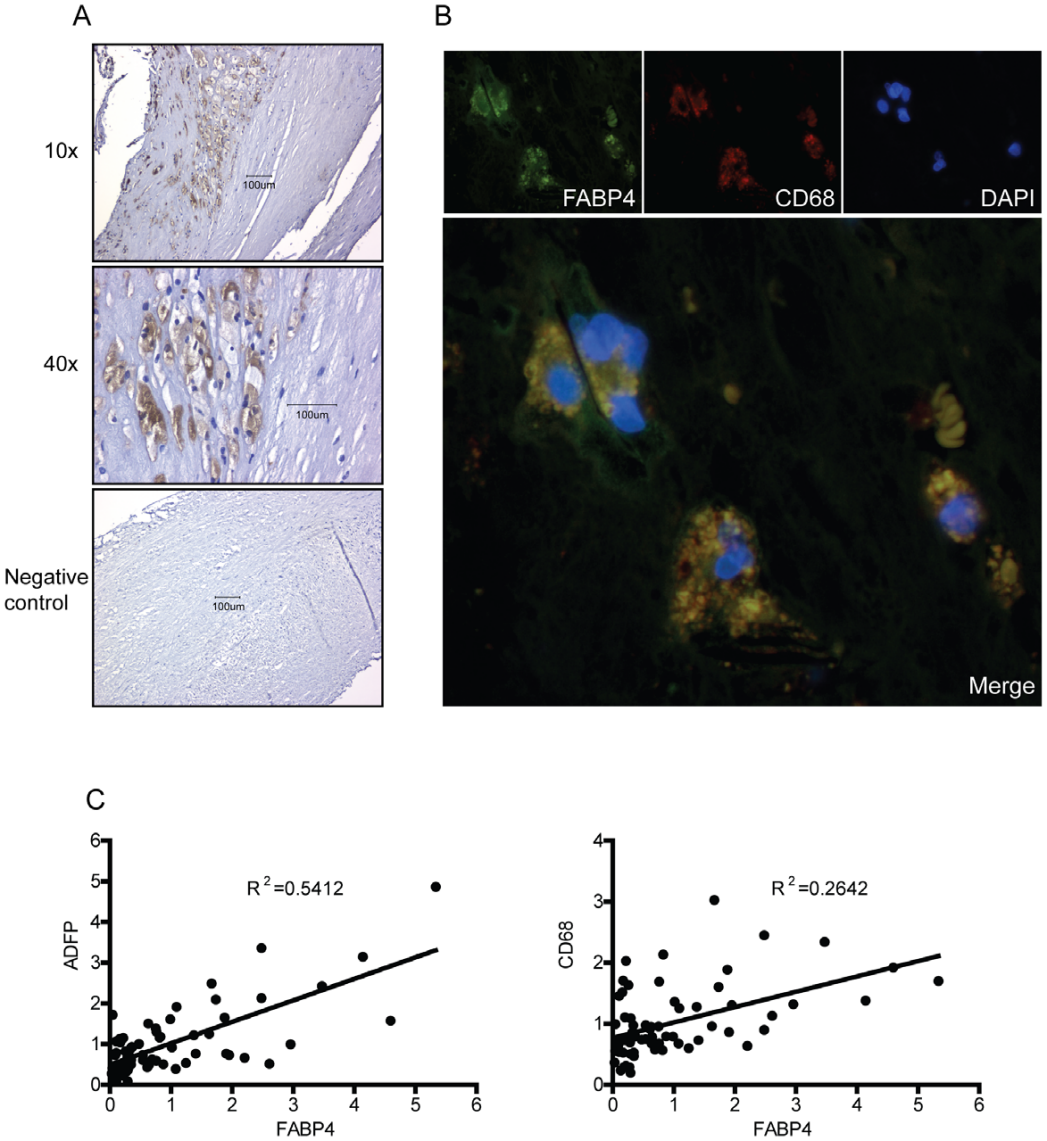
FABP4 is co-localized to macrophages within carotid atherosclerotic plaques. Panel **A** shows immunostaining of FABP4 in symptomatic carotid atherosclerotic plaques (n = 2) primarily located to macrophage-rich areas. Representative images obtained with 10x and 40x objective. Panel **B** shows double immunofluorescent staining of FABP4 (green fluorescence), CD68 (macrophages, red fluorescence) and nucleus (DAPI, blue fluorescence) from symptomatic carotid atherosclerotic plaques (n = 2). The lower panel is a merge of the three pictures above. Panel **C** shows the correlations of mRNA levels in atherosclerotic plaques between FABP4, ADFP and CD68, respectively.

We next examined the localization of FABP4 within plaques that were obtained from 2 patients with symptomatic carotid lesions (symptoms within the last month). Immunohistochemistry showed strong FABP4 immunostaining within areas rich in macrophages (Figure 3A), and the co-localization with macrophages was further supported by co-staining the plaques for both FABP4 and the macrophage marker CD68 (Figure 3B). To further examine the relationship between FABP4 and macrophages within the atherosclerotic lesion, we estimated the degree of macrophage infiltration by measurements of mRNA levels of the macrophage maker CD68 by real-time RT-PCR. Interestingly, mRNA levels of FABP4 were significantly correlated with mRNA levels of CD68 (R^2^ = 0.264, P<0.001) (Figure 3C). Moreover, mRNA levels of FABP4 were also significantly correlated with mRNA levels of the lipid droplet marker ADFP (R^2^ = 0.541, P<0.001) within the atherosclerotic lesions (Figure 3C).

### Oxidized LDL and platelets enhance FABP4 levels in THP-1 monocytes/macrophages

**Figure 4:**
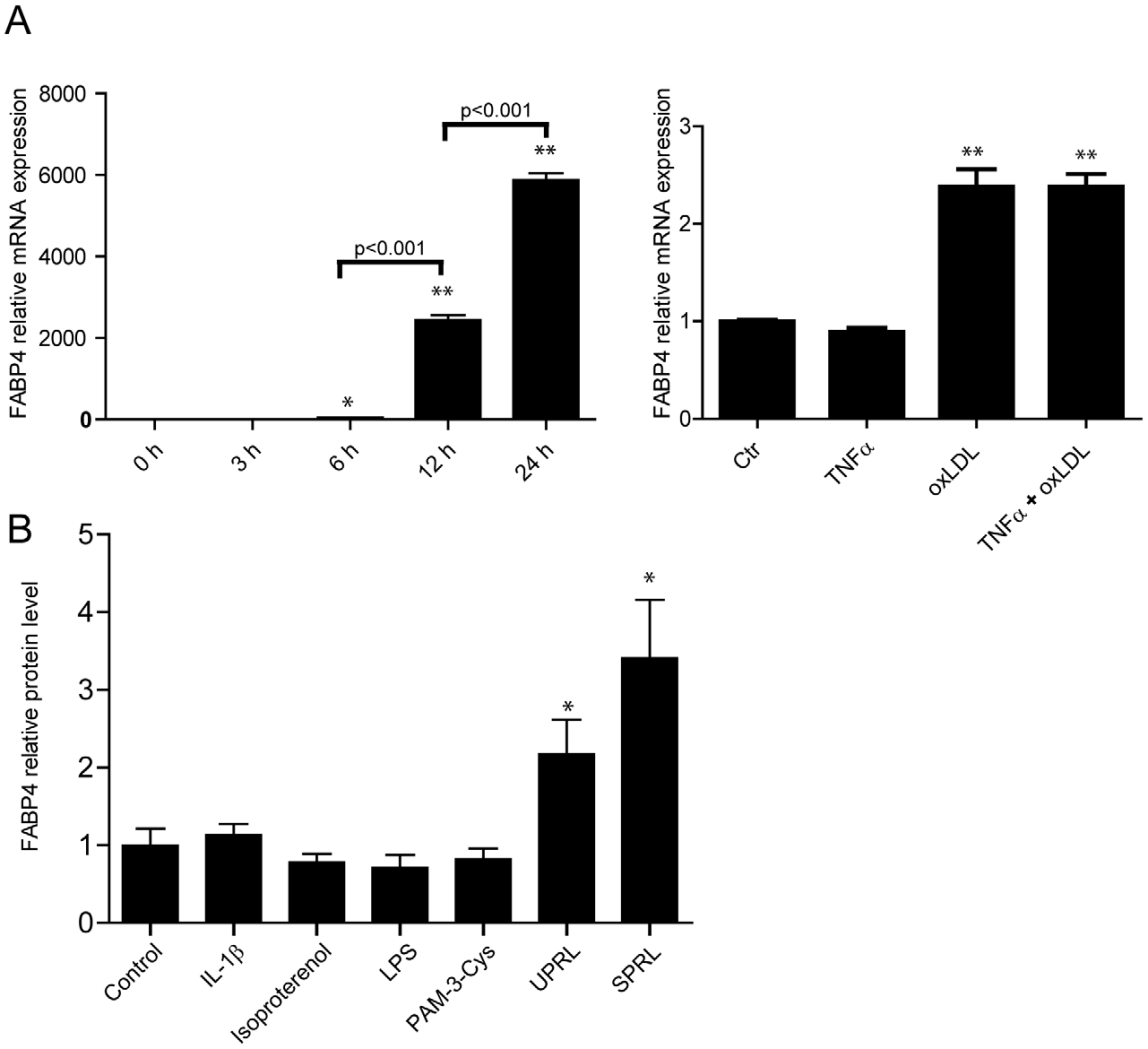
Oxidized LDL and platelets enhances FABP4 in macrophages. Panel **A** shows mRNA expression of FABP4 in THP-1 cells during PMA differentiation (left) and in THP-1 macrophages stimulated with oxidized LDL (20 µg/ml), TNFα (5 ng/ml) or both for 18 hours. mRNA levels were quantified with the use of real-time RT-PCR. The expression of β-actin was used as endogenous control. Panel **B** shows the release of FABP4 protein into the cell medium, as determined by enzyme immunoassay, in THP-1 monocytes that had been pre-incubated with rhTNFα (5 ng/ml) for 96 hours before being incubated with LPS (5 ng/ml), a TLR2 agonist (Pam3Cys, 1 µg/ml), isoproterenol (20 µM), rh-IL-1β (1 ng/ml) and platelet releasate from un-stimulated (UPRL) and thrombin-activated platelets (SPRL) for additional 20 hours. Data are mean ± SEM relative to values in un-stimulated cells (control). *p<0.05 and **p<0.001 versus control (or 0 hours [h] in panel A, left).

As FABP4 was primarily expressed in macrophages within the atherosclerotic lesion, we examined the ability of several stimuli with relevance to atherosclerosis to modify FABP4 expression in THP-1 monocytes/macrophages. Macrophage differentiation (Figure 4A-left) and oxLDL (20 µg/ml) (Figure 4A-right) markedly enhanced FABP4 mRNA expression in THP-1 macrophages. Inflammation could modulate FABP4 expression, but the addition of rhTNFα (5 ng/ml) to oxLDL stimulated THP-1 cells, carried out to mimic the inflammatory microenvironment within an atherosclerotic lesion, [16] did not influence the expression of FABP4 in these cells (Figure 4A-right). Thus, oxLDL induced FABP4 expression irrespective of co-incubation with TNFα. Moreover, releasate from both un-stimulated and in particular releasate from thrombin-activated platelets markedly enhanced the release of FABP4 protein in THP-1 monocytes that had been pre-incubated with rhTNFα (5 ng/ml) for 96 hours (Figure 4B). Although platelet releasate induced a release of FABP4 from THP-1 cells that had not been pre-incubated with TNFα, the increase was rather modest and with no difference between releasate from un-stimulated (1.6 +/− 0.05 -fold increase) and thrombin-activated (1.5 +/− 0.06 -fold increase) platelets. Thus, while the oxLDL-induced release of FABP4 in THP-1 monocytes seems to be independent of TNFα, the platelet-mediated release of this protein seems, at least partly, to depend on TNFα pre-incubation. In contrast to the effect of platelets, we found no effect of IL-1β, TLR2 and TLR4 activation and the β-adrenergic receptor agonist isoproterenol on FABP4 release from THP-1 cells that had been pre-incubated with rhTNFα (5 ng/ml) for 96 hours (Figure 4B).

### High plasma levels of FABP4 are associated with long-term mortality in patients with acute stroke

**Figure 5:**
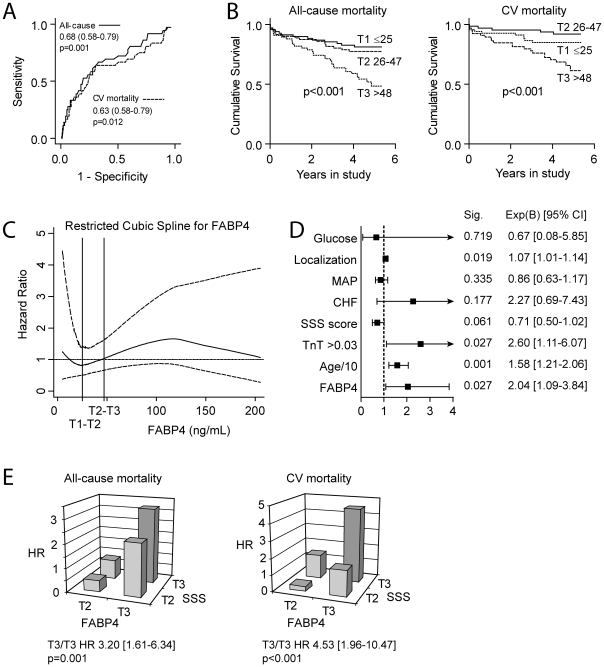
High plasma levels of FABP4 are associated with long-term mortality in patients with acute stroke. Panel **A** shows ROC curve analysis for the predictive value of FABP4 for all-cause and CV mortality. AUC and 95% CI are given. Panel **B** shows Kaplan-Meier curves with the cumulative incidence of all-cause and CV mortality during the entire study [median follow-up 4.4 years (interquartile range: 3.7 to 4.9 years)], according to tertiles of FABP4 at admission. Panel **C** shows the restricted cubic spline analysis of FABP4 in relation to all-cause mortality. Panel **D** shows multivariate analyses of FABP4 as an independent predictor of mortality in patients with acute stroke. *3^rd^ tertile vs. lower 2. Panel **E** shows the increase in hazard ratios (HR) for the prediction of all-cause and CV mortality when combing tertiles of FABP4 and SSS score (inverse for SSS, ie. increasing severity with higher tertiles). Tertile 1 was set as reference and the combination of T3 for both parameters is shown.

To further examine the relation of FABP4 to stroke, we examined determinants of FABP4 and the ability of baseline levels of FABP4 in plasma to predict long-term prognosis in 202 patients with acute ischemic stroke. Baseline characteristics of the study population are described in Table 2. In a multivariable stepwise linear regression model, the following were found to be independent predictors of FABP4 concentrations (1SD change): age (B = 0.02, SE = 0.01; p<0.001), gender (B = 0.84, SE = 0.13; p<0.001), log serum creatinine (B = 0.32, SE = 0.07, p<0.001), HDL cholesterol (B = −0.48, SE = 0.13, p<0.001) and SSS (B = −0.02, SE = 0.01, p = 0.002). At follow-up (median 4.4 years) 60 patients (29%) had died, 36 due to cardiovascular (CV) reasons. ROC analysis indicated that the level of FABP4 at admission had reasonable accuracy for the prediction of all-cause (AUC = 0.68; P <0.001; Figure 5A) and CV (AUC = 0.63; P = 0.012; Figure 5A) mortality at follow-up. Furthermore, Kaplan–Meier analyses using tertiles of FABP4 revealed a particularly high mortality rate in patients in the highest tertile (i.e., FABP4 > 48 ng/mL) (Figure 5B), with an unadjusted hazard ratio (HR) of 2.64 (95% CI = 1.59−4.38; P <0.001) for all-cause mortality and an unadjusted HR of 3.24 (1.67–6.29; P = 0.001) for CV mortality. Figure 5C shows the adjusted restricted cubic spline analysis of FABP4 and all-cause mortality indicating that the association with risk is non-linear. When analyzed as a continuous variable, the unadjusted HR and 95% CI for a 1 SD change in FABP4 was 1.61 (1.23–2.11) p = 0.001, while the adjusted HR showed no significant effect (HR 1.25 [0.87–1.78] p = 0.23).

In an adjusted model, this predictive value of the highest tertile of FABP4 was weakened (HR = 2.04 95% CI = 1.09−3.84; P = 0.027, Figure 5D) for all-cause mortality and not significant for CV mortality (HR = 1.49 95% CI = 0.62−3.59; P = 0.38). There were very few deaths within the first 30 days (n = 7), indicating that FABP4 levels at baseline may reflect the severity of the underlying atherosclerotic process rather than the severity of the index stroke (i.e., SSS). We therefore next looked at the combinations of tertiles of SSS score and FABP4 (Figure 5E) and found that the combination of high FABP4 and high stroke severity (ie. tertile 3) gave a high HR in relation to all cause (HR 3.20 95%CI = 1.61−6.34; P = 0.001) and CV (HR 4.53 95%CI =  1.96−10.47; P<0.001) mortality in univariate analysis. For all cause mortality, multivariate analysis indicated that the interaction term between the top tertiles of stroke severity and FABP4 was preferred over these factors alone with a similar HR as in univariate analysis (HR 3.27 95%CI = 1.46−7.34; P = 0.004). This was not the case in multivariate analysis of CV mortality where SSS score was preferred. Finally, we did not find that addition of FABP4 to the fully adjusted multivariate model had an effect on the prognostic discrimination for all-cause mortality (c-statistic without FABP4 0.741, with FABP4 0.747, p = 0.65) or CV mortality (c-statistic without FABP4 0.812, with FABP4 0.820, p = 0.37).

## Discussion

In the present study we have shown elevated FABP4 levels in patients with carotid atherosclerosis, both systemically and within the atherosclerotic lesion, with particularly high mRNA levels in carotid plaques from those with the most recent symptoms. Immunostaining of carotid plaques located FABP4 to macrophages, and plaque analyses showing a strong correlation between FABP4 *and* CD68 and ADRP, suggest that FABP4 could be a marker of lipid accumulation and macrophage infiltration within the lesion. Our *in vitro* findings also indicate a link between lipids and FABP4 expression within macrophages. Moreover, during acute ischemic stroke, high plasma levels of FABP4 were associated with total mortality and CV mortality during follow-up. The current study suggests that FABP4 may be related to carotid atherosclerosis, potentially reflecting plaque instability and poor outcome. However, future studies in larger stroke populations with differing etiology, reflecting the general stroke population, are needed to evaluate if FABP4 could be a biomarker for clinical risk stratification in patients with acute ischemic stroke.

Two recent studies have reported an association between the expression of FABP4 within carotid lesion and plaque instability, [9], [10] Peeters et al. [10] reported that high expression of FABP4 within the atherosclerotic lesion was predictive of the occurrence of adverse CV events. In the present study we extend these findings by showing that high plasma levels of FABP4 during acute ischemic stroke are associated with total and CV mortality during longitudinal follow-up. The ability of biomarkers to predict adverse outcome in atherosclerotic disorders may reflect their capacity to mirror several upstream pathways that are of pathogenic importance in these disorders. CRP is an established risk marker in CV, and it may be claimed that FABP4 is just another acute phase protein. However, although several biomarkers are inferior to CRP in patients with ischemic stroke, studies on other markers may be of importance to characterize the network of mediators that are involved in the development of this complex disorder.

The ability of oxLDL to enhance the expression of FABP4 in macrophages, as also shown in the present study, underscores a link between FABP4 levels and abnormal lipid metabolism during atherogenesis. Our data from plaque analyses further suggest that FABP4 could be a marker of lipid accumulation and macrophage infiltration within the lesion. The lack of correlation between plasma levels of FABP4 and lipid parameters may apparently seem in conflict with this finding. However, plasma data are more difficult to interpret, in particular as lipid status is modified by statins that was used by the majority of the patients. FABP4 has also been related to inflammatory responses within macrophages, and was recently also shown to regulate endoplasmatic reticulum homeostasis in these cells during atherosclerosis. [17] Somewhat surprisingly, we found no correlation between plasma levels of FABP4 and CRP, potentially reflecting that these markers may mirror different parts of the inflammatory arm of atherogenesis. Thus, it is tempting to hypothesize that the association of FABP4 with adverse outcome in stroke patients may reflect its ability to mirror upstreams pathways (e.g., lipid interaction with macrophages) that are only partly reflected by other biomarkers.

In the cross-sectional part of the study we found that while plasma levels of FABP4 did not differ between asymptomatic and symptomatic patients, plaque expression of FABP4 were higher in those with symptomatic lesions. It is possible that while plasma levels of FABP4 reflect the chronic atherosclerotic process, the expression of FABP4 within the atherosclerotic lesion will more directly be related to plaque instability and plaque inflammation at least in part reflecting the number of infiltrating macrophages within the lesion. This notion was also supported by the current data showing a strong correlation between FABP4 and CD68 within the atherosclerotic lesion. In the longitudinal study we found that the association between baseline levels of FABP4 and total mortality during follow-up mainly reflect an association between FABP4 and CV mortality. There were very few deaths within the first 30 days, further suggesting that FABP4 levels at baseline may reflect the severity of the underlying atherosclerotic process rather than the severity of the index stroke.

Our *in vitro* studies in THP-1 cells may suggest a link between platelet activation and enhanced expression of FABP4 within macrophages, at least partly dependent of pre-activation with TNFα. In contrast, we found no correlation between plasma levels of FABP4 and platelet counts or β-TG as a marker of platelet activation *in vivo.* Although these latter findings could have been influenced by the use of aspirin in the majority of patients, and although we can not exclude an interaction between platelets and monocytes/macrophages within the atherosclerotic lesion, further studies are needed to make any conclusion on the role platelet-monocyte/macrophage interaction in the regulation of FABP4 level.

In addition to be a marker of accelerated atherosclerosis, FABP4 may also be an important mediator in this process. Deletion of FABPs, including FABP4, in adipocytes has been reported to result in reduced expression of inflammatory cytokines in macrophages. [18] Moreover, an orally active small-molecule inhibitor of aP2 (the mice homologue to FABP4) has been found to be an effective therapeutic agent against severe atherosclerosis and type 2 diabetes in mouse models. [19] Genetically and chemical modification of aP2 have also been shown to protect against the deleterious effects of hyperlipidemia in macrophages. [17] Thus, the importance of FABP4 as a mediator seems to involve adipocytes, macrophages and their interaction. The combined properties of being a mediator as well as a marker of enhanced atherogenesis may further support its potential role as a biomarker in stroke and related disorders. However, although the association between FABP4 and mortality in multivariate cox regression as well as other data in this manuscript suggest that FABP4 may be linked to enhanced atherogenesis, we did not find that addition of FABP4 to the fully adjusted multivariate model had an effect on the prognostic discrimination for all-cause mortality as assessed by c-statistics. Our interpretation is that FABP4, at least at present, is not a “stand alone” biomarker which can be used clinically to identify patients at risk, but our findings suggest that studying circulating FABP4 in larger stroke populations could be of interest

The current study has some limitations. The exclusion criteria represent both strength and a weakness. By excluding patients with atrial fibrillation, overt ischemic heart disease or patients with onset of stroke symptoms >7 days before admission, all states characterized by an increased inflammatory component, it seems likely that the FABP4 levels do not merely reflect inflammatory responses in concurrent disorders. However, the exclusion of many patients also represents a weakness since the relevance of our findings to the general stroke population remains unknown, and unfortunately, we lack data on the clinical characteristics of the screened patients that were not included. Moreover, the number of patients that were included in the cross-sectional study was rather low, and data from this part of the study should be interpreted with caution. Furthermore, the lack of data on HbA1c as an important risk factor, and a more reliable marker on glucose metabolism than fasting glucose, in patients with acute stroke is a limitation of this part of the study. Also, correlation analyses should be interpreted with caution, and further mechanistic studies as well as studies in larger stroke populations are needed to elucidate the role of FABP4 in atherosclerotic disorders. Nonetheless, our findings may suggest a link between FABP4 and atherogenesis and plaque instability in patients with carotid atherosclerosis and acute ischemic stroke.
